# MicroRNA-7 regulates endocrine progenitor delamination and endocrine cell mass in developing pancreatic islets

**DOI:** 10.1016/j.isci.2024.110332

**Published:** 2024-06-20

**Authors:** Eva Kane, Tracy C.S. Mak, Mathieu Latreille

**Affiliations:** 1 MRC Laboratory of Medical Sciences, Du Cane Road, London W12 0NN, UK

**Keywords:** Cell biology, Molecular biology, Physiology

## Abstract

β-cell replenishment in patients with diabetes through cadaveric islet transplantation has been successful; however, it requires long-term immunosuppression and suitable islet donors are scarce. Stepwise *in vitro* differentiation of pluripotent stem cells into β-cells represents a viable alternative, but limitations in our current understanding of *in vivo* islet endocrine differentiation constrains its clinical use. Here, we show that microRNA-7 (miR-7) is highly expressed in embryonic pancreatic endocrine progenitors. Genetic deletion of the miR-7 gene family in endocrine progenitors leads to reduced islet endocrine cell mass, due to endocrine progenitors failing to delaminate from the epithelial plexus. This is associated with a reduction in neurogenin-3 levels and increased expression of Sry-box transcription factor 9. Further, we observe that a significant number of endocrine progenitors lacking miR-7 differentiate into ductal cells. Our study suggests that increasing miR-7 expression could improve efficiency of *in vitro* differentiation and augment stem cell-derived β-cell terminal maturity.

## Highlights

• miR-7 is expressed in delaminating progenitors in developing mouse pancreas
• miR-7 KO in mouse islet endocrine progenitors leads to reduced mature cell mass
• Endocrine progenitors lacking miR-7 do not delaminate, redirecting to a ductal fate
• miR-7 plays a pivotal role in driving efficient β-cell differentiation

## Graphical abstract

**Figure:**
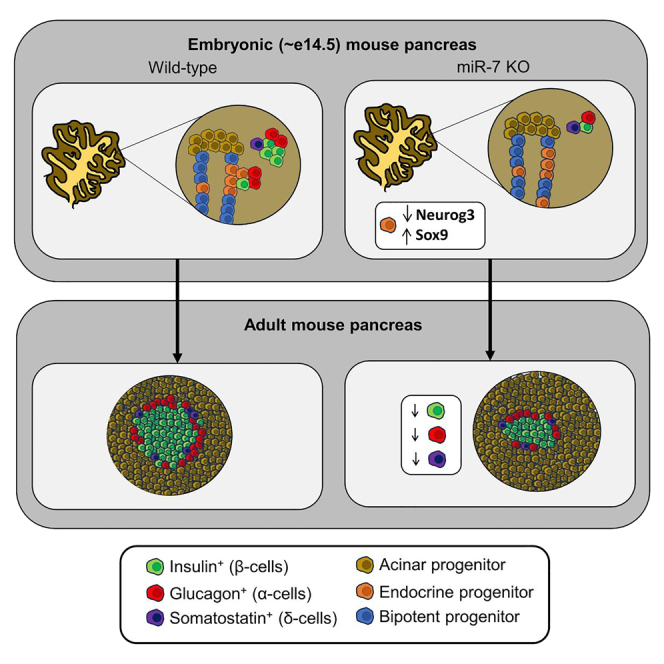

## Introduction

Diabetes is a global health crisis projected to increase to 700 million cases by 2045.^1^ Diabetes is caused by loss of glucose homeostasis, which in healthy individuals is controlled by the islets of Langerhans (clusters of endocrine cells in the mammalian pancreas).^2^ β-cells respond to increased circulating glucose by secreting insulin,^3^ while α-cells secrete glucagon when blood glucose is low.^4^^,^^5^ Insulin and glucagon act on peripheral tissues to lower or increase glycemia, respectively.^6^^,^^7^^,^^8^^,^^9^ Somatostatin, secreted by δ-cells, fine-tunes insulin and glucagon activity.^10^^,^^11^ Although there are three main subtypes of diabetes (Type 1, Type 2, and Maturity Onset Diabetes of the Young), in all cases the primary proximal cause of hyperglycemia is absolute or relative deficit of functional β-cells. Consequently, functional β-cell replacement therapy is a potential gold standard of treatment.

Cadaveric intraportal islet transplantation, in which healthy donor islets from deceased individuals are infused via the hepatic portal vein and engraft into the liver, can confer long-term endogenous insulin production and glycemic stability to patients with diabetes.^12^^,^^13^ Although insulin independence is only retained for over two years in 31% of patients, patients with refractory diabetes generally remain protected against severe hypoglycaemia,^13^ improving their quality of life.^14^ However, transplantable islets are expensive to harvest^15^ and scarce.^16^ Long-term immunosuppression is also required, causing side effects.^13^ Consequently, the procedure is only considered for refractory Type 1 diabetes with no reasonable alternative treatment. A potential alternative for β-cell replacement therapy is stem cell-derived β-cells (SC-β-cells), generated by stepwise exposure of pluripotent stem cells to small molecules, mimicking endogenous islet development signals.

Understanding pancreas development has informed progress toward SC-β-cell derivation. Pancreas development is conserved in vertebrates, with murine models being the best studied. Around embryonic day (e) 8.5 of mouse development,^17^ pancreatic buds begin to arise from posterior foregut endoderm^18^^,^^19^ in response to signals from the notochord^20^^,^^21^ and lateral plate mesoderm.^22^^,^^23^ These buds are composed of pancreatic progenitors (PPs) expressing transcription factor pancreatic and duodenal homeobox 1 (Pdx1).^24^^,^^25^ PPs give rise to a branching plexus of epithelial cells, which will become the adult pancreatic duct.^26^ The plexus is regionalised into the tip domain, giving rise to acinar cells, and trunk domain, giving rise to ductal and endocrine cells via a bipotent intermediate progenitor (BP).^27^ Neurogenin-3 (Neurog3), a basic-helix-loop-helix transcription factor, is transiently expressed in select BPs, inducing endocrine progenitor (EP) specification.^28^^,^^29^ In response to cues including the Notch pathway^30^^,^^31^ and mechanical signals,^26^^,^^32^^,^^33^ EPs delaminate from the plexus while differentiating to hormone^+^ endocrine cell precursors, ultimately forming islets.^34^

MicroRNAs (miRNAs) are short (∼22 nucleotide) non-coding RNAs playing a crucial role in islet development.^35^ miRNAs inhibit gene expression via imperfect base-pairing interactions with sequences in the 3′ untranslated region (UTR) of target mRNAs, leading to translational repression and accelerated mRNA degradation.^36^^,^^37^^,^^38^^,^^39^^,^^40^ RNase III endonuclease Dicer is required for miRNA biogenesis.^41^ Conditional deletion of the *Dicer* gene in developing pancreas leads to islet defects including reduction in pancreatic EP number and postnatal β-cell apoptosis,^35^^,^^42^ yet little is known about individual miRNAs regulating islet development. Global genetic miR-375 inactivation in mice leads to hyperglycemia due to increased α-cell and decreased β-cell mass resulting from impaired proliferation.^43^^,^^44^ Selective re-expression of miR-375 in β-cells normalizes α- and β-cell mass.^45^ miR-7 is also involved in pancreas development. In mammals, miR-7 is transcribed from three highly conserved genes.^46^^,^^47^^,^^48^ *miR-7a1* is embedded within an intron of *Hnrnpk* (chromosome 13), while *miR-7a2* and *miR-7b* are intergenic genes located on chromosome 7 and 17, respectively.^49^ In mice and humans, the three functionally redundant miR-7 miRNAs share identical seed sequences. miR-7 is highly expressed in pancreatic endocrine development, coinciding with the peak of *Neurog3* expression (∼e14.5 in mice).^50^^,^^51^ Conserved genomic elements displaying promoter activity upstream of the *miR-7a2* gene are targeted by Neurog3,^52^ suggesting a possible causal relationship between miR-7a2 and Neurog3 expression. Fluorescent *in situ* hybridization (FISH) indicates that miR-7 colocalizes with Neurog3 and islet hormones in the epithelial plexus trunk at e13.5.^53^ miR-7 knockdown in 48-h cultured e12.5 mouse dorsal pancreatic explants β-cell mass at the expense of ε-cells.^53^ Conversely, e10.5 miR-7 knockdown via the intrauterine fetal heart injection of antisense morpholinos led to reduced β-cell mass.^51^ Overall, miR-7’s role during islet endocrine cell specification is unclear. Nonetheless, *in vitro* experiments show that miR-7 mimic delivery during SC-β-cell differentiation leads to increased glucose-stimulated insulin secretion (GSIS).^54^ In adulthood, miR-7 regulates insulin granule exocytosis^55^ and β-cell identity.^56^

To understand the role of the miR-7 gene family in pancreatic endocrine specification, we conditionally ablated the expression of the three miR-7 genes in EPs. Our results identify a critical role for miR-7 in regulating endocrine progenitor delamination and islet endocrine cell mass and resolve the existing conflict around the role of miR-7 in islet development, paving the way for targeted application to *in vitro* derivation.

## Results

### microRNA-7 expression in delaminating endocrine progenitors

**Figure 1:**
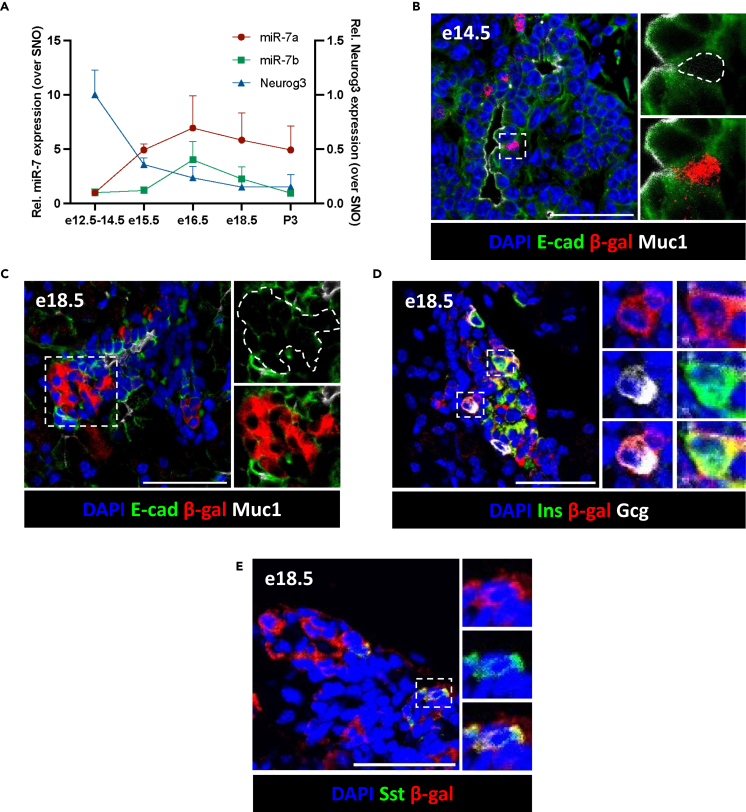
miR-7a2 is expressed in the endocrine lineage of developing mouse pancreas (A) RT-qPCR of miR-7a1 and miR-7a2 (miR-7a), miR-7b and Neurog3 in mouse pancreatic buds at e12.5–18.5 and P3. Data are mean ± SEM. n = number of embryos/pups, *n* = 4 for e12.5–14.5 and P3, *n* = 7 for e15.5–18.5, (B and C) Representative image of a miR-7a2^LacZ/LacZ^ mouse pancreatic bud at (B) e14.5 and (C) e18.5, stained with DAPI (blue), or with antibodies against E-cad (green), β-gal (red) and Muc1 (white), (D and E) Representative image of a miR-7a2^LacZ/LacZ^ mouse pancreatic bud section at e18.5, stained with DAPI (blue), and with antibodies against β-gal (red) and (D) insulin (Ins) (green) and glucagon (Gcg) (white), or (E) somatostatin (Sst) (green). LacZ gene product β-gal reports endogenous miR-7a2 expression. Scale bar = 50 μm.

To define the miR-7 gene family expression profile during mouse development, pancreatic buds were isolated and subjected to RT-qPCR using Taqman probes for miR-7a and miR-7b (Figure 1A). This indicated that miR-7a (miR-7a1 and/or miR-7a2) expression increases between e12.5 and e15.5 following Neurog3 expression’s peak, rising to its highest level at e16.5 before declining neonatally. miR-7b is expressed later ∼ e15.5–e16.5, but its levels also decrease in neonatal mice. Given that most islet miR-7 expression is derived predominantly from the miR-7a2 locus,^45^ we corroborated these findings using a miR-7a2 reporter mouse (miR-7a2^LacZ/LacZ^) in which a LacZ gene is controlled by the endogenous miR-7a2 upstream regulatory region. Immunofluorescence staining of β-galactosidase (β-gal) was observed in scattered cells of e14.5 mouse embryonic pancreas (Figure 1B). β-gal^+^ cells co-localized with E-cadherin^+^ cells surrounding the lumen of the epithelial plexus, including in mucin 1 (Muc1)^+^ cells. Apical narrowing of β-gal^+^Muc1^+^ cells is morphologically consistent with delaminating EPs at this timepoint.^34^ β-gal^+^Muc1^-^ cells are consistently E-cad^+^, indicating that they are epithelial cells originating from the PP lineage and not the surrounding mesenchyme. These observations suggest that miR-7a2 is expressed in cells within the epithelial plexus and recently delaminated EPs. Consistent with this, e18.5 β-gal^+^ cells are found in clusters close to, but not in contact with, the Muc1^+^ plexus lumen (Figure 1C). At this stage, β-gal is co-expressed with insulin (Figure 1D), glucagon (Figure 1D), and somatostatin (Figure 1E), marking β-, α-, and δ-cell precursors, respectively. Hormone^+^/β-gal^-^ cells were not observed, suggesting that miR-7a2 expression is restricted to endocrine precursors at this timepoint. In adult pancreatic sections, β-gal immunoreactivity is found in β- and δ-cells and is no longer detected in α-cells (Figures S1A–S1C).

### Loss of microRNA-7 in endocrine progenitors leads to increased hormone^+^ precursor number but reduced mature endocrine cell mass

**Figure 2:**
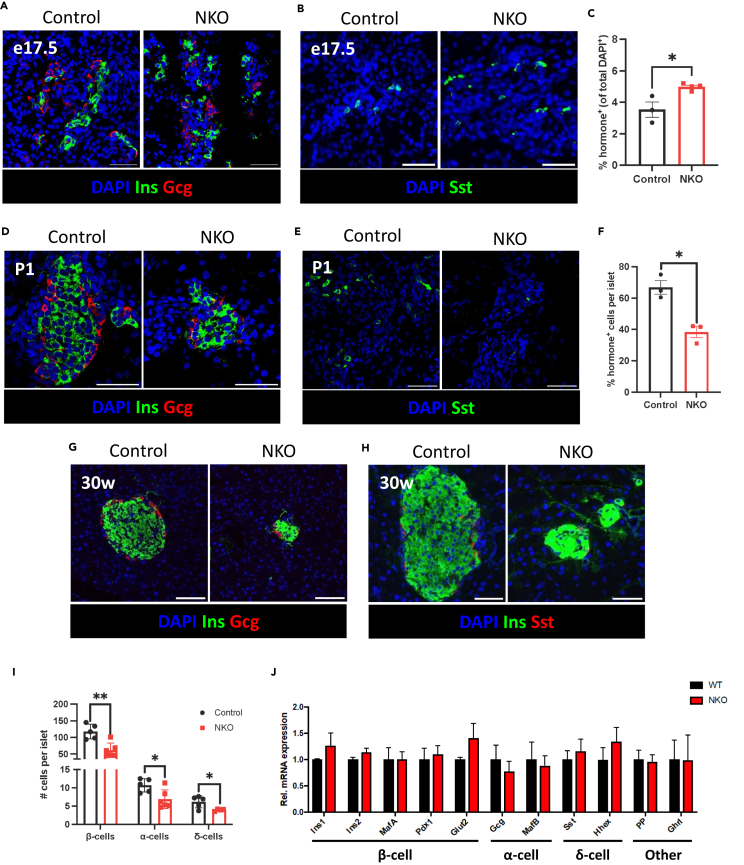
Loss of endocrine progenitors in NKO mice leads to increased volume of hormone+ precursors within the epithelial plexus, but reduced mature delaminated endocrine cell mass in adulthood (A and B) Representative images of pancreatic buds from control and NKO e17.5 embryos, stained with DAPI (blue) and with antibodies against insulin (Ins) (green), glucagon (Gcg) (red), or somatostatin (Sst) (green) as indicated, (C) Quantification of the percentage of cells (DAPI+) that are Ins+, Gcg+, or Sst+ in e17.5 pancreatic bud sections, (D and E) Representative images of pancreas from control and NKO neonatal (P1) pups stained with DAPI (blue), and with antibodies against insulin (Ins) (green), and glucagon (Gcg) or somatostatin (Sst) (green) as indicated, (F) Quantification of the number of cells per islet that are Ins+, Gcg+, or Sst+ in neonatal pancreas sections, (G and H) Representative images of pancreatic islets from control and NKO adult islets, stained with DAPI (blue), and with antibodies against insulin (Ins) (green), and glucagon (Gcg) (red), or somatostatin (Sst) (red), as indicated (I) Quantification of β-, α-, and δ-cell mass in adult islets, (J) RT-qPCR of mature islet endocrine identity markers, for islets isolated from control and NKO mice (*n* = 4/group). Control mice were Cre^–^ littermates of NKO mice. Scale bar = 50 μm. n = number of animals. Data are mean ± SEM and were analyzed using Student’s t test (C, F, I) or two-way ANOVA (J). ∗*p* < 0.05, ∗∗*p* < 0.01.

To elucidate the role of the miR-7 gene family in islet development, we genetically inactivated *miR-7a1*, *miR-7a2* and *miR-7b* genes in EPs using the Cre-LoxP system. Conditional miR-7-floxed mice were crossed with mice expressing Cre recombinase controlled by the Neurog3 promoter region,^55^^,^^57^ generating mice with miR-7 gene deletion in Neurog3-expressing cells (NKO) and Cre^−^ controls. Genetic inactivation of miR-7 genes was confirmed via RT-qPCR of isolated islets from NKO mice compared to control littermates (Figure S2). First, we examined the effect of miR-7 deletion in hormone^+^ endocrine precursors, given miR-7 expression in these cells. Immunofluorescence in e17.5 NKO embryonic pancreas revealed an increased proportion of cells expressing one of the three most abundant islet hormones – insulin, glucagon, or somatostatin (Figures 2A–2C). This corresponded to a trend toward an increase in the percentage of insulin^+^, glucagon^+^, and somatostatin^+^ cells individually (Figure S3A). Conversely, similar analyses in neonatal (P1) NKO and control pups revealed a trend toward fewer β-, α-, and δ-cells (Figures 2D–2F). Immunofluorescence staining of insulin, glucagon, and somatostatin in pancreas sections from adult (30-week) NKO and control littermates revealed that reduced endocrine cell mass persists into adulthood, with a marked reduction in overall islet size (Figures 2G–2I). RT-qPCR of these islets revealed no significant difference in relative mRNA expression levels of islet endocrine cell identity markers such as MafA, MAF BZIP transcription factor B (MafB), haematopoietically expressed homeobox (Hhex), or pancreatic polypeptide (Figure 2J), suggesting that mature endocrine identity and proportional fate allocation is maintained in mutant mice. Aligning with reduced endocrine cell mass, NKO mice showed progressive random-fed hyperglycemia for three weeks (Figure S3B) and mild glucose intolerance when challenged with glucose (Figure S3C), despite maintaining similar body weight to controls (Figure S3D). Together, our findings reveal that the miR-7 gene family is required for the determination of islet endocrine cell mass.

### Apoptosis and proliferation rates are unaffected in embryonic and postnatal mice

**Figure 3:**
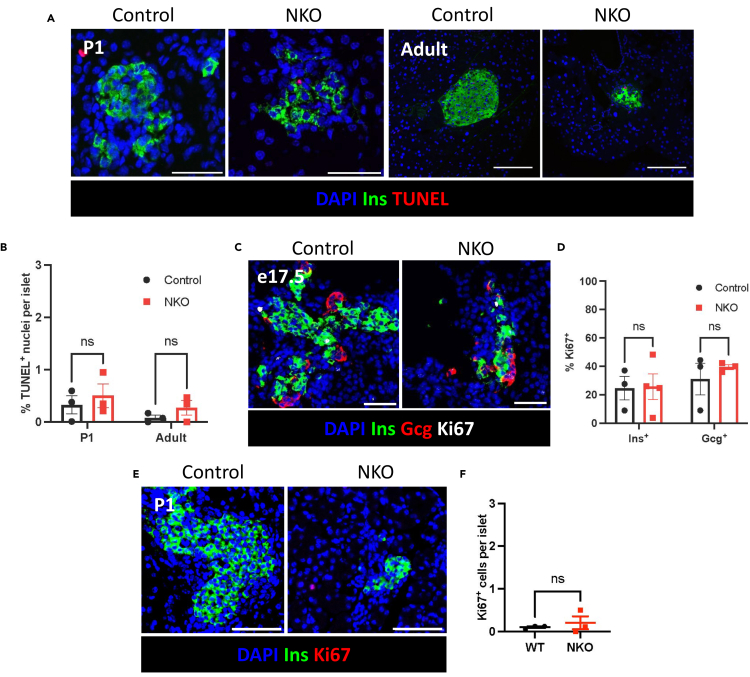
Pancreatic apoptosis and proliferation rates are unaffected in embryonic and postnatal NKO mice (A) Representative images of neonatal (P1) and 30-week old (adult) pancreatic islets, stained with DAPI (blue), and with antibodies against insulin (Ins) (green), and labeled for TUNEL (red), (B) Quantification of the percentage of TUNEL^+^ nuclei per islet, (C) Representative images of pancreatic buds from e17.5 control and NKO embryos, stained with DAPI (blue), and with antibodies against insulin (Ins) (green), glucagon (Gcg) (red) and Ki67 (white), (D) Quantification of the percentage of Ins^+^Ki67^+^ and Gcg^+^Ki67^+^ cells per pancreas section in e17.5 pancreatic buds, (E) Representative images of the pancreas from neonatal pups stained with DAPI (blue), and with antibodies against insulin (Ins) (green), and Ki67 (red), (F) quantification of the percentage Ki67^+^ cells per islet in neonatal pancreas. Scale bar = 50 μm. n = number of animals. Data are mean ± SEM and were analyzed using Student’s t test. ns *p* < 0.05.

Given the reduction in endocrine cell mass in NKO mice between e17.5 and adulthood, we assessed apoptosis using terminal deoxynucleotidyl transferase biotin-dUTP nick end labeling (TUNEL)^+^ at P1 and in adult (30-week) islets. This analysis did not show a significant difference in the percentage of TUNEL^+^ islet nuclei between NKO mice and control littermates (Figures 3A and 3B). Moreover, we did not find any change in α- or β-cell proliferation rate in NKO islets at e17.5, shown by the percentage of insulin^+^ or glucagon^+^ cells which co-express Ki67^+^ (Figures 3C and 3D). Similarly, at P1, there was no significant difference between NKO mice and controls in the percentage of Ki67^+^ cells per islet (Figures 3E and 3F). We were unable to investigate the proliferation rate in δ-cells due to antibody incompatibility.

### Lineage tracing reveals failure of endocrine progenitors to delaminate from epithelial plexus

**Figure 4:**
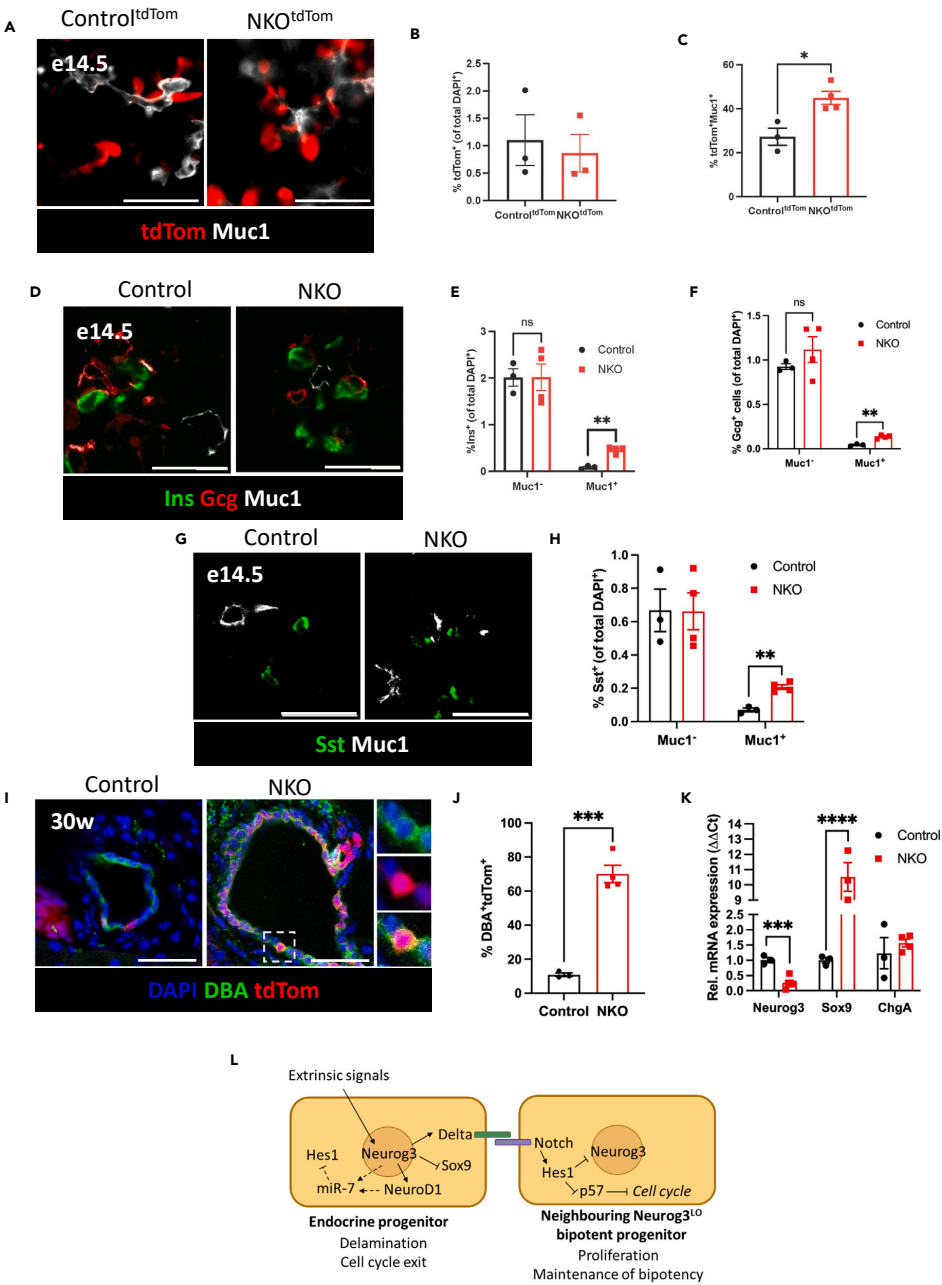
Lineage tracing reveals failure of endocrine progenitors to delaminate from epithelial plexus in NKO mice (A) Representative images of e14.5 pancreas, endogenously expressing tdTomato (tdTom) (red) and stained with an antibody against mucin-1 (Muc1) (white), (B) Quantification of the percentage of tdTom^+^ cells per embryonic pancreas section, (C) Quantification of the percentage of tdTom^+^ cells co-expressing Muc1, (D) Representative images of e14.5 pancreas, stained with antibodies against insulin (Ins) (green), glucagon (Gcg) (green), and Muc1 (white), (E) Quantification of Ins^+^/Muc1^-^ and Ins^+^/Muc1^+^ cells per embryonic pancreas section, (F) Quantification of Gcg^+^/Muc1^-^ and Gcg^+^/Muc1^+^ cells per embryonic pancreas section, (G) Representative images of e14.5 pancreas stained with antibodies against somatostatin (Sst) (green) and Muc1 (white), (H) Quantification of Sst^+^/Muc1^-^ and Sst^+^/Muc1^+^ cells per embryonic pancreas section, (I) Representative images of adult (30-week old) pancreatic duct, endogenously expressing tdTom and stained with DAPI (blue) and dolichos bifluoros agglutinin (DBA) (green), (J) Quantification of DBA^+^ cells co-expressing tdTom per pancreatic section, (K) RT-qPCR of tdTom^+^ cells in e15.5 embryonic pancreas, (L) Proposed model of miR-7 action in delaminating endocrine progenitors. Scale bar = 50 μm. n = number of animals. Data are mean ± SEM and were analyzed using Student’s t test (C–D, K) or two-way ANOVA (F–G, I, L). ns *p* > 0.05, ∗*p* < 0.05, ∗∗*p* < 0.01, ∗∗∗*p* < 0.01, ∗∗∗∗*p* < 0.001.

To better understand how the genetic inactivation of miR-7 in EPs leads to reduced islet endocrine cell mass postnatally, we performed genetic lineage tracing to monitor EP fate in NKO mice. To accomplish this, we crossed NKO mice with mice harboring a LoxP-STOP-LoxP-tdTomato cassette, resulting in the generation of NKO^tdTom^ and control (Control^tdTom^) mice. In both groups of mice, cells initiating Neurog3 expression and their daughter cells are irreversibly labeled with tdTomato. FAC-sorted tdTomato^+^ cells from control^tdTom^ islets expressed higher levels of miR-7 and islet endocrine markers and ∼10-fold lower expression of mesenchymal marker TGF-β compared with tdTomato^−^ (Figures S4A and S4B), indicating that our labeling system specifically marked endocrine cells. Adult pancreatic sections from NKO^tdTom^ and control^tdTom^ mice also show a complete overlap of tdTomato expression with the endocrine marker chromogranin A (ChgA) (Figure S4C). At e14.5, we found that while there is a similar proportion of tdTomato^+^ cells in both control^tdTom^ and NKO^tdTom^ pancreas, a higher percentage of tdTomato^+^ cells in NKO^tdTom^ embryonic pancreas expressed Muc1 on their apical surface compared with control^tdTom^ mice (Figures 4A–4C), indicating that fewer EPs have delaminated from the epithelial plexus. Furthermore, we found that e17.5 pancreatic sections from NKO animals show an increase in the proportion of α-, β-, and δ-cell precursors co-expressing Muc1, whereas the percentage of insulin, glucagon, or somatostatin^+^Muc1^-^ cells is unchanged (Figures 4D–4H). This suggests that the increase in hormone^+^ precursors in e17.5 NKO embryos is due to impaired delamination from the epithelial plexus. Next, we traced the fate of the hormone^+^ precursors which fail to delaminate in adult mice using fluorescently labeled dolichos biflorus agglutinin (DBA), which binds to N-acetylgalactosamine on the surface of pancreatic ductal cells. In adult pancreatic sections, we found a significant increase in the percentage of tdTomato^+^/DBA^+^ cells in NKO^tdTom^ ducts compared to control^tdTom^ (Figures 4I and 4J). These observations suggest that EPs in NKO mice fail to delaminate from the epithelial plexus and acquire a ductal fate instead. Supporting this, we found that FAC-sorted tdTomato^+^ cells from e15.5 NKO^tdTom^ embryos showed decreased Neurog3 expression compared to control^tdTom^ littermates, whereas levels of Sox9, a ductal marker, were higher in tdTomato^+^ cells from NKO^tdTom^ than control^tdTom^ embryos (Figure 4K). Overall, our findings indicate that miR-7 promotes the delamination of EPs during the development and specification of pancreatic endocrine cells.

## Discussion

Using a miR-7 reporter model, we report that miR-7a2 is expressed in delaminating EPs and precursors of α-, β-, and δ-cells. Furthermore, we demonstrate an *in vivo* knockout mouse model of the miR-7 family in Neurog3^+^ cells. We find that NKO mice display increased EPs and EP-derived hormone^+^ precursors within the epithelial plexus during embryogenesis yet reduced islet endocrine cell mass postnatally, despite no increase in apoptosis or decrease in proliferation compared to controls. Genetic lineage tracing with tdTomato confirmed that a high proportion of EP-derived cells remain in the epithelial plexus of NKO embryos and, consequently, the adult pancreatic duct. We attribute this to insufficient EP delamination leading to ductal cell fate acquisition. Taken together, our results indicate that miR-7 promotes EP delamination during mouse pancreas development. To our knowledge, this study uncovers the first miRNA gene family regulating EP delamination and reveals an additional regulatory mechanism complementing the action of Neurog3^58^ and transcriptional regulators of delamination, including the co-repressor Grg3.^59^

TdTomato^+^ cells in e15.5 NKO^tdTom^ mice have reduced Neurog3 and increased Sox9 expression compared with controls. Sox9 is a BP marker and is required for the transient transcriptional induction of Neurog3 but downregulated during endocrine differentiation directly by Neurog3 transcriptional repression, forming a negative feedback loop.^60^ Sox9 also activates hairy and enhancer of split-1 (Hes1), a component of the Notch signaling pathway and a transcriptional repressor of the Neurog3 gene.^61^ Consequently, BPs must 'escape' Sox9-and Hes1-mediated Neurog3 repression in order to become EPs^62^ (Figure 4L). Differential Notch signaling inputs contribute to the modulation of progenitor differentiation.^31^ Intermediate Notch pathway activity is required for endocrine differentiation whereas high Notch pathway activity in EPs results in Sox9 maintenance and the redirection of Neurog3^+^ progenitors toward a ductal fate,^31^ highlighting the fundamental role played by Notch dosage in driving BP differentiation toward either endocrine and ductal lineages. The downregulation of Neurog3 and the upregulation of Sox9 in NKO^tdTom^ embryos suggested that miR-7 could be integrated in this circuit during endocrine differentiation.

Interestingly, the *Drosophila* homolog of Hes1, *hairy*, is a confirmed *in vivo* target of miR-7^63^ and there is a highly conserved miR-7 binding site in the 3′ UTR of mammalian Hes1 (Table S1). Direct inhibition of Hes1 by miR-7 has also been shown *in vitro* in mammalian cell culture.^64^ We hypothesize that miR-7 could be integrated within a regulatory circuit reinforcing Neurog3 activity through the post-transcriptional repression of Hes1. Supporting this conjecture, the reallocation of Neurog3^+^ to a ductal fate in the NKO model is comparable to the effect of constitutive Notch activation in Neurog3^+^ cells.^65^ Neurog3 activation of the *miR-7a2* gene has been shown *in vitro*, and NeuroD1, a downstream effector of Neurog3, also transactivates the functionally redundant miR-7b gene.^53^ We propose that the integration of these transcriptional circuits with miR-7-mediated Hes1 repression could form a coherent feedforward loop, fine-tuning organ patterning by reinforcing BP maturation into EPs (Figure 4L). This is also supported by data from Dicer KO in PPs, in which there is increased Hes1 expression at e12.5 followed by a significant reduction in pancreas size, ductal branching, and Neurog3^+^ cell number.^35^ Further research is required to demonstrate direct and physiologically relevant repression of Hes1 by miR-7 at this conserved site in bipotent and endocrine progenitors specifically.

In recent years, there has been growing interest in reproducing the mechanical microenvironment created by intrinsic and extrinsic developmental cues to enhance the efficiency of *in vitro* derivation protocols and the functionality of derived SC-β-cells. López-Beas et al. demonstrated that miR-7 expression peaks at day 5 of *in vitro* SC-β-cell differentiation, corresponding to the appearance of definitive endoderm.^54^ Interestingly, delivery of miR-7 mimics at day 14, analogous to the stage at which EP delamination occurs, increases SC-β-cell insulin content and GSIS.^54^ Modulation of cytoskeletal organization with the actin depolymerizing agent latrunculin-A, mimicking the mechanical microenvironment of delamination, has proven a successful strategy for increasing endocrine induction in 2D *in vitro* derivation protocols.^33^ Our results in NKO mice suggest that the functional inhibition of miR-7 at day 5 of differentiation protocols could improve the yield of functional SC-β-cells by preventing the premature initiation of EP delamination and differentiation pathways. Combining miR-7 mimics with pharmacological actin depolymerization agents at a later point in differentiation could help to trigger delamination, promoting endocrine lineage and simultaneously reducing ductal fate allocation. Further, 3D protocols that employ scaffolds to mimic morphological cues^66^ could be supplemented with miR-7 delivery to synergistically promote endocrinogenesis. Since miR-7 is uniformly expressed in α-, β-, and δ-cells during development, manipulating its levels could be particularly beneficial for facilitating the *in vitro* derivation of whole islets as opposed to single β-cells.

Our finding that the genetic deletion of miR-7 leads to reduced islet endocrine cell mass supports the findings of Nieto et al.,^51^ who found that miR-7 knockdown at e10.5 via the fetal heart injection of antisense morpholinos led to reduced β-cell mass and that e12.5 pancreatic buds cultured for 5 days with antisense morpholinos display decreased insulin expression (Ins1 and Ins2). These results conflict with findings from Kredo-Russo et al.^52^ indicating that miR-7 knockdown in 48-h cultured mouse dorsal explants does not result in decreased β-cell mass. It is therefore possible that the specific experimental set-up used by Kredo-Russo et al. for *ex vivo* culture of their explants (only 48-h treatment vs. 5 days for Nieto et al.) may have differentially affected the differentiation of the genetically manipulated cells. Alternatively, the level of miR-7 inhibition reached in both transplant studies may explain the different results. Further work may be needed to resolve this discrepancy.

In our previous work, we demonstrated that a β-cell-specific knockout of the gene encoding miR-7a2 using a rat insulin promoter (RIP)-Cre driver has no impact on β-cell mass and led to improved glucose tolerance by increasing insulin secretion.^55^ This seems to conflict with our finding that an endocrine progenitor-specific miR-7 gene family knockout using a Neurog3-Cre driver leads to reduced β-cell mass and adult-onset hyperglycemia. As the three members of the miR-7 gene family are functionally redundant,^55^ it may be that there is sufficient expression of miR-7 from *miR-7a1* and *miR-7b* to facilitate normal endocrine delamination. Further, RIP-Cre-mediated *miR-7a2* deletion in β-cells is likely to occur at a later developmental timepoint than in the NKO mouse model reported here, missing the developmental window in which miR-7 is required to promote endocrine progenitor delamination. Finally, miRNA function varies depending on which mRNA targets are being expressed in the cell of interest at a particular timepoint. As such, a single miRNA gene could impact glucose control differently when inactivated during embryonic development than in adult mice. Our results in NKO mice, in tandem with the previous data from Latreille et al.,^55^ strongly suggest that miR-7 function in islet endocrine cells varies depending on developmental state.

Results presented here further our understanding of miR-7 function during islet development and resolve conflicting observations previously obtained *in vitro*.^51^^,^^53^ We report a previously unknown role for the miR-7 gene family in EP delamination and specification. We speculate that the modulation of miR-7 levels could improve the efficiency of *in vitro* SC-β-cell derivation for β-cell replacement therapy in diabetes.

### Limitations of the study

Although we provide strong evidence for our findings, some limitations were inherent in the execution of this study. It would be valuable to examine the co-localization of β-gal with Neurog3 in miR-7a2-LacZ embryonic pancreas; however, we were unable to identify a specific Neurog3 antibody giving a consistent and reliable immunofluorescence signal under conditions compatible with available β-gal antibodies. Nonetheless, the EP-like pattern of miR-7a2 expression in scattered cells of the epithelial plexus is supported by FISH-based expression profiling of miR-7 in mouse embryos^53^ and human fetal pancreas,^50^ supporting the notion that miR-7a2 is indeed expressed in Neurog3^+^ EPs. Previous work suggested that miR-7 is expressed in α-, β-, and δ-cells of adult human pancreas,^50^^,^^67^ which is inconsistent with our observations indicating exclusive expression in β- and δ-cells in mice, suggesting that the miR-7 expression pattern in mature α-cells may differ between mouse and human. Finally, while we show that mature islet endocrine cells in NKO mice express functional identity markers at similar levels to controls, *in vitro* GSIS would be required to confirm that NKO β-cells are indeed fully functional and that the only source of hyperglycemia is reduced islet endocrine cell mass. We did not perform this experiment as it would be extremely technically challenging due to the small size of NKO islets.

## STAR★Methods

### Key resources table

**Table:** 

REAGENT or RESOURCE	SOURCE	IDENTIFIER
**Antibodies**		

Chicken anti-β-galactosidase (1:100)	Abcam	Cat # AB9361; RRID: AB_307210
Rabbit anti-chromogranin A (1:500)	Immunostar	Cat # 20085; RRID: AB_572227
Mouse anti-E-cadherin (1:100)	BD Bioscience	Cat # 610181; RRID: AB_397580
Rabbit anti-glucagon (1:500)	Millipore	Cat # AB932; RRID: AB_2107329
Guinea pig anti-insulin (1:2)	Dako	Cat # A0654; RRID not available
Rabbit anti-Ki67 (1:200)	Abcam	Cat # AB16667; RRID: AB_302459
Armenian hamster anti-mucin1 (1:100)	Invitrogen	Cat # MA5-11202; RRID: AB_11000874
Rabbit anti-somatostatin (1:500)	Dako	Cat # A0566; RRID: AB_2688022
Goat anti-guinea pig AlexaFluor 488 (1:500)	Invitrogen	Cat # A11073; RRID: AB_2534117
Goat anti-guinea pig AlexaFluor 647 (1:500)	Invitrogen	Cat # A21450; RRID: AB_2535867
Goat anti-chicken AlexaFluor 594 (1:200)	Invitrogen	Cat # A11042; RRID: AB_2534099
Goat anti-rabbit AlexaFluor 488 (1:500)	Invitrogen	Cat # A11008; RRID: AB_143165
Goat anti-rabbit AlexaFluor 568 (1:500)	Invitrogen	Cat # A11011; RRID: AB_143157
Goat anti-rabbit AlexaFluor 647 (1:500)	Invitrogen	Cat # A21244; RRID: AB_2535812
Goat anti-Armenian hamster AlexaFluor 488 (1:200)	Jackson	Cat # 127-545-160; RRID: AB_2338997
Goat anti-Armenian hamster AlexaFluor 647 (1:200)	Jackson	Cat # 127-605-160 RRID: AB_2339001
Donkey anti-mouse fluorescein isothiocyanate (FITC) (1:200)	Jackson	Cat # 715-095-151; RRID: AB_2335588

**Chemicals, peptides, and recombinant proteins**		

Triton	Sigma	T8787-250ML
Goat serum	Sigma	D9663-10ML
Donkey serum	Sigma	G9023-10ML
Optimal cutting temperature (OCT) compound	VWR	361603E
Histopaque 1077	Sigma	10771-6X100ML
Liberase TM Research Grade (Collagenase I + II)	Sigma	05401127001
RNAlater Stabilization Solution	Invitrogen	AM7020
RPMI 1640 powder for 10L	Invitrogen	51800035
TRIzol	Invitrogen	10296010
Kapa Sybr Fast quantitative PCR MasterMix optimized for LightCycler® 480	Kapa Biosystems	KK4611
TaqMan® Fast Advanced MasterMix	Applied Biosystems	4444556
Trypsin	Promega	V5113
Fetal bovine serum (FBS)	Sigma	F7524
Penicillin-streptomycin	Thermo Fisher	15140122
Bovine serum albumin (BSA)	Sigma	A3059-100g
Vectashield Mounting Medium	Vector Labs	H-1000

**Critical commercial assays**		

*In Situ* Cell Death Detection Kit (TMR Red)	Roche	12156792910
High-Capacity cDNA Reverse Transcription kit (with RNase inhibitor)	Thermo Fisher	4374966
TaqMan® MicroRNA Reverse Transcription Kit	ThermoFisher	4366596

**Experimental models: Organisms/strains**		

C57BL/6J mice	Charles River	RRID:MGI:3028467
Gt(ROSA)26Sor^tm14(CAG-tdTomato)Hze/J^	The Jackson Laboratory	RRID:IMSR_JAX:007908
miR-7a1^fl/fl^ mice	ETH Zurich^55^	Not available
mir-7a2^fl/fl^ mice	ETH Zurich^55^	Not available
miR-7b^fl/fl^ mice	ETH Zurich^68^	Not available
miR-7a2^LacZ/+^ mice	MMRRC	RRID:MMRRC_034648-JAX
Neurog3-Cre C1Able/J mice	The Jackson Laboratory	RRID:IMSR_JAX:005667

**Software and algorithms**		

CellProfiler v3.1.8	Broad Institute	https://cellprofiler.org
GraphPad Prism 9	GraphPad Software	http://www.graphpad.com
ImageJ	National Institute of Health	https://imagej.nih.gov/ij
Zen v2.6	Zeiss	https://www.zeiss.com/microscopy/en/products/software/zeiss-zen.html

**Other**		

DAPI		
*Dolichos bifluoros agglutinin* (1:25)	VectorLabs	Cat # FL-1031
Polysine slides	SLS	MIC3050
Superfrost slides	SLS	MIC3040
Peel-A-Way Square S-22 embedding molds	Sigma	E6032
Round-bottomed tube with cell stainer cap	Corning	352235
70 μm cell strainer	BD Falcon	352350

### Resource availability

#### Lead contact

Further information and requests for reagents may be directed to the lead contact Mathieu Latreille (matlat@yahoo.com).

#### Materials availability

This study did not generate new unique reagents.

#### Data and code availability

All data will be shared by the lead contact upon request. This paper does not report original code. Any additional information required to reanalyze the data reported in this paper is available from the lead contact upon request.

### Experimental model and study participant details

#### Mouse strains and husbandry

ARRIVE guidelines were used for animal experiment designing and reporting. All work was carried out in accordance with the UK Animals (Scientific Procedures) Act (1986) and approved by the Imperial College Animal Welfare and Ethical Review Body and UK Home Office (licence 70/8967). All mice were maintained in a C57BL/6J background, on a 12-h light/dark cycle with *ad libitum* access to water and standard laboratory chow RM3 diet (SDS), in specific pathogen-free barrier facilities.

C57BL/6J mice (RRID:MGI:3028467) were purchased from Charles River. miR-7a2^LacZ/+^ mice (RRID:MMRRC_034648-JAX),^69^ were obtained from the MMRRC repository. miR-7a1^fl/fl^, mir-7a2^fl/fl^ and miR-7b^fl/fl^ mice were previously generated (RRIDs not available).^55^^,^^68^ Neurog3-Cre C1Able/J mice (Neurog3-Cre) (RRID:IMSR_JAX:005667)^57^ and Gt(ROSA)26Sor^tm14(CAG-tdTomato)Hze/J^ mice (Rosa26/tdTomato^stop fl/fl^) (RRID:IMSR_JAX:007908)^70^ were purchased from The Jackson Laboratory. Male mice were used preferentially to reduce variability due to estrous cycle and sex differences in endocrine differentiation.^71^ For embryological experiments, both male and female mice were used due to practical considerations.

Timed pregnancies were set up by housing one stud male with 1–3 dams. Dams were checked for vaginal plugs in the morning between 7.30 and 8.30 a.m.; if present, the plugged dam was separated and that day was considered to be e0.5. Random-fed blood glucose measurements were taken weekly at the same time via tail venesection using Contour Next EZ glucometer test strips (Bayer). Intraperitoneal glucose tolerance tests (IPGTTs) were performed in the morning following an overnight 18-h fast using 2 g/kg glucose.

### Method details

#### Histology

Mice were euthanised and the pancreas was dissected out and fixed in 4% PFA at 4°C. For embryos, the uterus was removed, and embryos were dissected out and euthanised. For paraffin embedding, tissue was fixed overnight, dehydrated in a Tissue-Tek VIP Tissue Processor (Sakura) and embedded in paraffin. Blocks were cut into 4 μm sections on a microtome (Thermo Fisher), and mounted on polysine slides (SLS). Sections were rehydrated in decreasing concentrations of ethanol and xylene and, if needed, antigen retrieval performed in 10 mM sodium citrate pH 6.0 in a decloaking pressure chamber. Sections were permeabilised at room temperature in 0.1% Triton (Sigma) in PBS (perm buffer [PB]) and blocked at room temperature in blocking buffer (BB) containing 5% serum matching secondary antibody host/1% bovine serum albumin (BSA) in PB. BB was exchanged for primary antibody diluted in BB and incubated in a humidified chamber overnight at 4°C. Slides were washed in PB and incubated at room temperature with an appropriate secondary antibody diluted in BB. Nuclei were stained with 1/10,000 4′,6-diamidino-2-phenylindole (DAPI) in PBS. Slides were washed in PB, and mounted on glass cover slips (SLS) in Vectashield Mounting Medium (Vector Labs). For optimal cutting temperature (OCT) embedding, tissues were fixed for 4 h and transferred to 30%/PBS sucrose (Fisher) overnight followed by 15% sucrose/50% OCT (VWR) for 1 h. Tissues were embedded in OCT in Peel-A-Way Square S-22 embedding molds (Sigma), frozen on dry ice and stored at −80°C. Samples were cut into 10–15 μm sections and mounted on SuperFrost slides (SLS). Immunofluorescence staining performed as above with PBS washes. Terminal deoxynucleotidyl transferase dUTP nick end labeling (TUNEL) was performed using the *In Situ* Cell Death Detection Kit (TMR Red) (Roche) per manufacturer’s instructions.

#### Imaging

For two-week and adult pancreas, 3–5 levels (100 μm apart) were taken for quantitative analysis. Serial sections were taken from embryonic and neonatal pancreas and quantitative analysis performed using sections at least three sections apart from one another.

Representative images and images of embryonic pancreas for quantitative analysis were taken with a TCS SP5 confocal microscope (Leica, DM6000 CS). Gain, exposure, and laser power were set using a relevant control slide and kept consistent within experiment. For all other quantitative analyses, an AxioScan Z1 slide scanner (Zeiss) was used. Representative images were processed using ImageJ.

#### Islet isolation

Islets were isolated following pancreas perfusion via the bile duct of euthanised mice with 0.2 mg/mL Liberase TM Research Grade (Collagenase I + II) (Sigma). Pancreata were incubated at 37°C before inactivation by addition of Roswell Park Memorial Institute (RPMI) medium supplemented with 10% FBS (Sigma), then filtered through a mesh to remove undigested tissue and resuspended in Histopaque 1077 (Sigma) layered with RPMI medium. Following centrifugation at 2200 rpm without braking, islets were collected with a Pasteur pipette and filtered through a 70 μm strainer into a 6-well plate containing RPMI/FBS supplemented with penicillin and streptomycin (Thermo Fisher). Islets were handpicked intro progressive wells of the 6-well plate to isolate. Isolated islets were washed with PBS, resuspended in RNAlater Stabilization Solution (Invitrogen), and frozen on dry ice before being stored at −80°C.

#### RNA analysis

RNA was extracted from tissue using TRIzol. mRNA reverse transcription (RT) was performed using High-Capacity cDNA Reverse Transcription Kit (with RNase inhibitor) (Thermo Fisher) per manufacturer’s instructions. RT was performed in a thermal cycler. cDNA was diluted with deionised water to a level appropriate for expected transcript abundance (typically 1:10–1:100). RT-qPCR was performed using Kapa Sybr Fast quantitative PCR MasterMix optimised for LightCycler 480 (Kapa Biosystems) per manufacturer’s instructions. Relative mRNA concentration was calculated using ΔΔCt or standard curves. Data were normalised by 36B4. RT-qPCR was performed in a LightCycler 480 (Roche). miRNA RT was performed using the TaqMan MicroRNA Reverse Transcription Kit (Thermo Fisher) per manufacturer’s instructions, using custom RT primers for miR-7a (miR-7a1 and –7a2) and miR-7b. RT-qPCR was performed using TaqMan Fast Advanced MasterMix (Applied Biosystems) per manufacturer’s instructions.

#### Fluorescence-activated cell sorting (FACS)

Embryonic pancreas was dissociated using 0.05% trypsin in PBS incubated at 37°C. Samples were resuspended in 1 mL FACS buffer (2% FBS and 2mM EDTA in PBS) and transferred to a round-bottomed tube with cell strainer cap (Corning). TdTomato^+^ cells were isolated using a BD FACSAria III Cell Sorter and RNA extraction was performed as described above.

### Quantification and statistical analysis

#### Image quantification

Images of islets obtained using the Axioscan Z1 slide scanner were extracted using Zen software. All quantified islet images were analyzed using CellProfiler v3.1.8 or by manual counting. All analyses were done using at least three sections per animal. All positive cells per section were counted.

#### Statistical anlaysis

Data are represented as mean ± standard error of the mean (SEM). Statistical significance was tested using two-tailed Student’s t test or two-way analysis of variance (ANOVA) for grouped analyses, unless otherwise indicated in figure legend (normal distribution assumed). Number of biological replicates (n; number of animals) is indicated in figure legends. All analyses were performed in GraphPad Prism 9. Grubb’s test for outliers was applied to all datasets and significantly outlying data points were excluded. Statistical significance was defined as *p* ≤ 0.05, ∗*p* ≤ 0.05, ∗∗*p* ≤ 0.01, ∗∗∗*p* ≤ 0.001, ∗∗∗∗*p* ≤ 0.0001.
