# Supplementary material for: MicroRNA-7 regulates endocrine progenitor delamination and endocrine cell mass in developing pancreatic islets
Source: iScience. 2024 Jun 20;27(7):110332. doi: 10.1016/j.isci.2024.110332 (PMC11269303; doi:10.1016/j.isci.2024.110332)
Supplement: Document S1. Figures S1–S4 and Table S1 [file mmc1.pdf]

iScience, Volume *27*

Supplemental information

MicroRNA-7 regulates endocrine progenitor delamination and endocrine cell mass in developing pancreatic islets

Eva Kane, Tracy C.S. Mak, and Mathieu Latreille

Supplementary Material

Figure S1. miR-7a2 expression is restricted to insulin (Ins)-expressing β-cells and somatostatin (Sst)-expressing δ-cells in mature mouse islets, Related to Figure 1

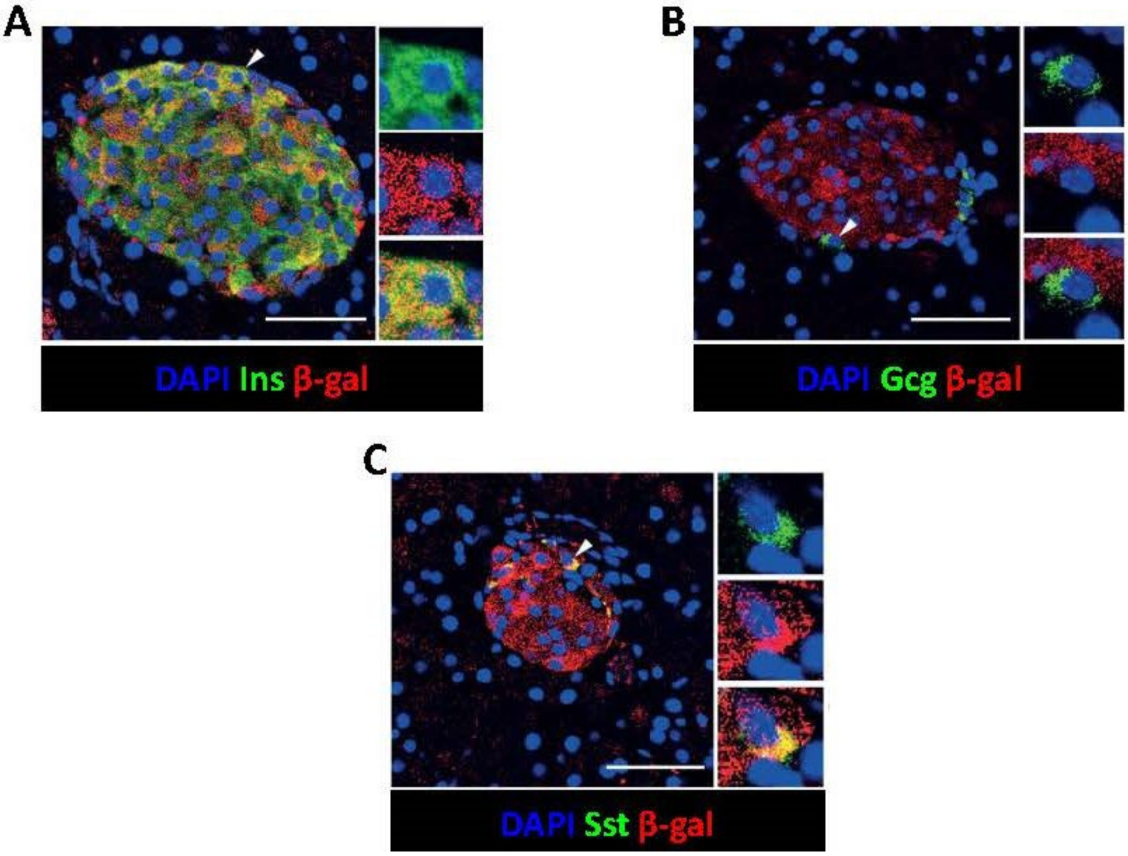

Representative images of miR-7a2LacZ/LacZ adult mouse pancreas stained with DAPI (blue), or with antibodies against β-gal (red), and (A) Ins, (B) glucagon (Gcg), and (C) Sst (green). LacZ gene product β-gal reports endogenous miR-7a2 expression. Scale bar = 50 µm.

Figure S2. Validation of miR-7 knockout in adult NKO mouse islets, Related to Figures 2–4

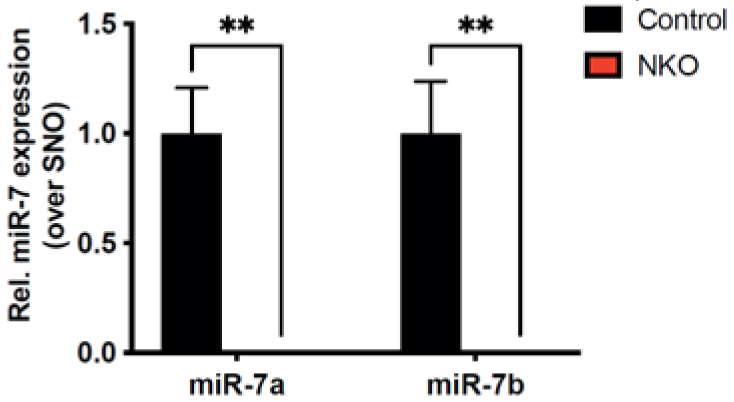

RT-qPCR of isolated pancreatic islets from adult (30 week old) control and NKO mice for miR-7a (miR-7a1 and miR-7a2) and miR-7b. Control mice were Cre^{-} littermates. n = number of animals; n = 5/group. Data are mean ± SEM and were analysed using two-way ANOVA. **p<0.01.

Figure S3. Adult NKO mice develop hyperglycaemia and glucose intolerance There is a non-significant trend towards an increase in insulin (Ins)⁺, glucagon (Gcg)⁺, and somatostatin (Sst)-expressing hormone⁺ precursors in embryonic pancreas, Related to Figure 2–4

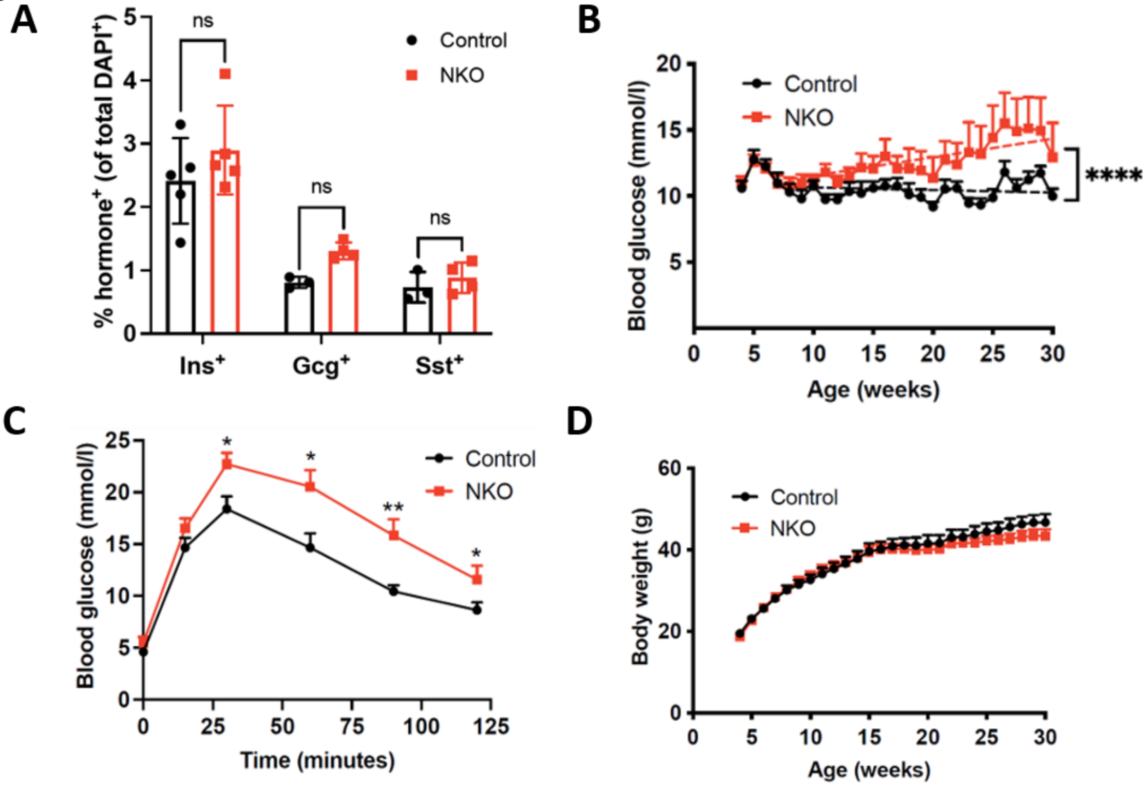

(A) Quantification of the percentage of cells (DAPI⁺) in e17.5 pancreatic bud sections that are Ins⁺, Gcg⁺ and Sst⁺ individually. Scale bar = 50 µm. Control mice were Cre⁻ littermates. Data are mean ± SEM and were analysed using two-way ANOVA. ns $p>0.05$. (B) Random-fed blood glucose (mmol/l) taken weekly from weaning to 30 weeks of age. Linear regression indicates progressively increasing glycaemia in NKO mice over time and a significant difference in slope between control and NKO groups, (C) IPGTT showing glycaemia (mmol/l) following IP injection of a 2 g/kg bolus of glucose. Control mice were Cre⁻ littermates of NKO mice. (D) Body weight of control and NKO mice, taken weekly from weaning (3 weeks) to 30 weeks of age. n = number of animals. n = 9/group (B-D). Data are mean ± SEM. *$p<0.05$, **$p<0.01$, ***$p<0.001$, ****$p<0.0001$.

Figure S4. Validation of lineage tracing in Control^{tdTom} and NKO^{tdTom} mice, Related to Figure 4

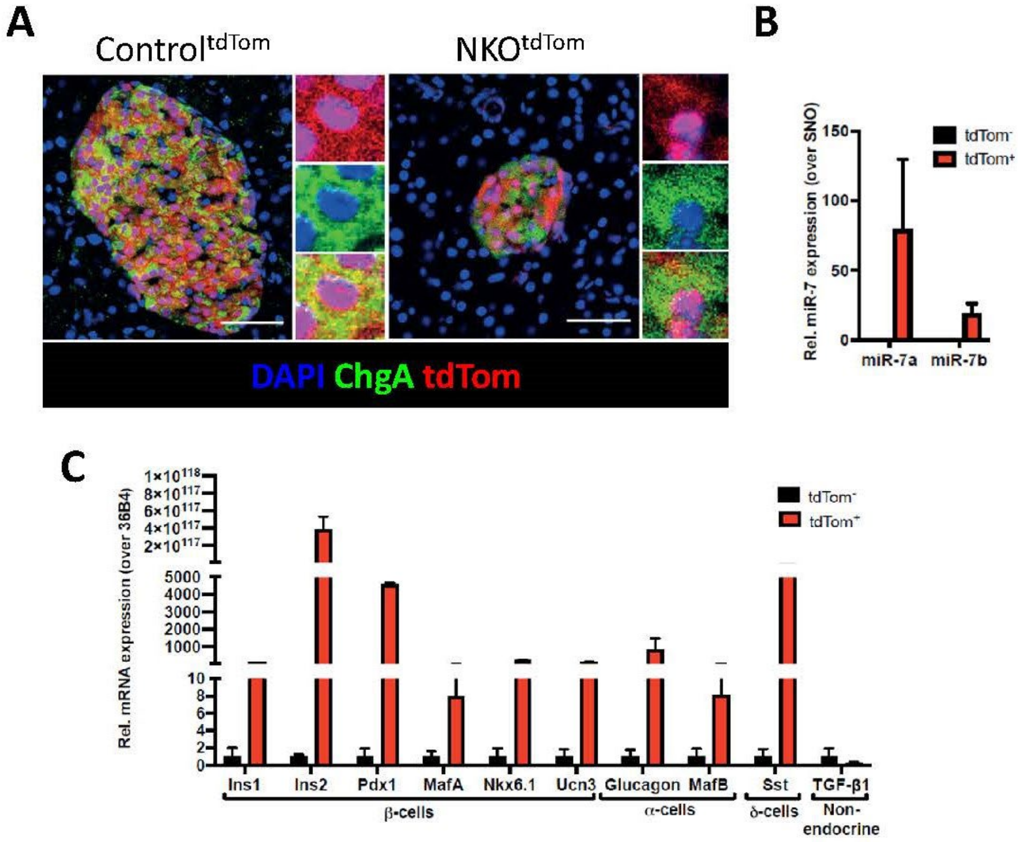

(A) Representative images of pancreatic islets from adult (15-week old) Neurog3-Cre x tdTomato (tdTom) (control^{tdTom}) and NKO x tdTom (NKO^{tdTom}) pancreatic sections, endogenously expressing tdTom (red) and stained with DAPI (blue) and antibodies against chromogranin A (ChgA) (green), (B) RT-qPCR of tdTom$^{+/-}$ cells sorted by FACS from isolated pancreatic islets of adult control^{tdTom} mice for miR-7a1 and miR-7a2 (mir-7a) and miR-7b, (C) RT-qPCR of tdTom$^{+/-}$ cells sorted by FACS from isolated pancreatic islets of adult control^{tdTom} mice for mRNA transcribed from genes involved in defining islet endocrine or non-endocrine cell identity, as indicated. n = number of animals (n = 2/group). Data are mean ± SEM.

Table S1. A conserved miR-7 binding site (red) is found in the gene encoding Hes1, Related to Figure 4

	**Sequence**
**miR-7-5p**	3’ UTUUGUUUUAGUGAUCAGAAGGU 5’
**Rat Hes1 3’ UTR**	5’ GAAAAATGCTCTTAAAATATCTTCCT 3’
**Mouse Hes1 3’ UTR**	5’ TGAAAATGCTCTTAAAATATCTTCCT 3’
**Human Hes1 3’ UTR**	5’ TGAAAATGCTCTTAAAATATCTTCCT 3’

Seed sequence highlighted in red.
